# Supplementary material for: Decoupling the origins of irreversible coulombic efficiency in anode-free lithium metal batteries
Source: Nat Commun. 2021 Mar 4;12:1452. doi: 10.1038/s41467-021-21683-6 (PMC7933276; doi:10.1038/s41467-021-21683-6)
Supplement: Supplementary file 1 — Supplementary Information [file 41467_2021_21683_MOESM1_ESM.pdf]

Decoupling the origins of irreversible coulombic efficiency in anode-free lithium metal batteries

Chen-Jui Huang[1], Balamurugan Thirumalraj[1], Hsien-Chu Tao[1], Kassie Nigus Shitaw[1], Hogiartha Sutiono[1], Tesfaye Teka Hagos[2], Tamene Tadesse Beyene[1], Li-Ming Kuo[1], Chun-Chieh Wang[3], She-Huang Wu[2], Wei-Nien Su[2], Bing Joe Hwang[1,3,4*]

[1]Department of Chemical Engineering, National Taiwan University of Science and Technology, Taipei, Taiwan

[2]Graduate Institute of Applied Science and Technology, National Taiwan University of Science and Technology, Taipei, Taiwan

[3]National Synchrotron Radiation Research Center (NSRRC), Hsinchu, Taiwan

[4]Sustainable Energy Development Center, National Taiwan University of Science and Technology, Taipei, Taiwan

In-operando transmission X-ray microscopy for Li plating and stripping

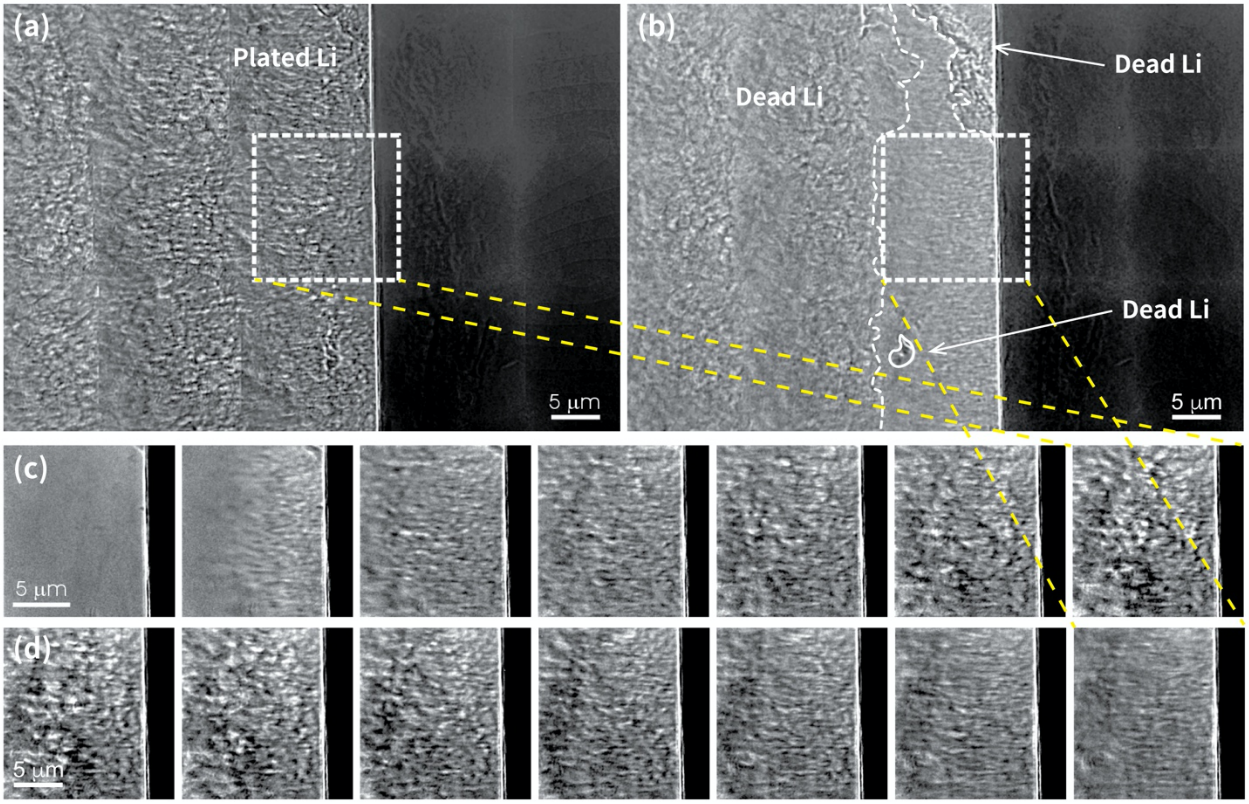

Supplementary Fig. 1. In-operando TXM measurement for Li plating/stripping. Mosaic images of the Cu surface **a.** after Li deposition and **b.** Li stripping under the current density of 2 mA cm^{-2} with 1M $LiPF_6$ in EC:DEC as the electrolyte. **c.** From left to right are the selected images showing the Li growth during the deposition process in the inset of **a**. **d.** From left to right are the selected images showing the Li stripping during the dissolution process in the inset of **b**. All the images share the same scale bar with the length of 5 μm.

Fraction of dead Li^0 and SEI Li^+ in Li//Cu cell determined by the TGC method. Due to the fact that it is unable to separate the fraction of dead Li and SEI simply from the electrochemical results of Li//Cu cell. A precise quantification method like TGC measurement[1] is required for determining the fractions of dead Li and SEI in each cycle of the Li//Cu

cells. Thus, we have measured the proportion of dead Li and SEI in each cycle from one to five cycles of Li//Cu cells by the TGC method (Supplementary Fig. 2). The results of the TGC measurements and electrochemical tests show that after the 1st cycle, the irr-CEs of Li//Cu cell are quite comparable in the subsequent cycles, with the fraction of subsequent SEI gradually increased in each cycle. This can be explained as the instability of SEI and the subsequent fracture due to dendrite formation[2]. To conclude, since the proportion of dead Li and subsequent SEI needs to be quantified by TGC measurements, the two origins of irr-CE are combined in the proposed protocol and denoted as dead Li + sub. SEI. As for the difference between the 1st cycle irr-CE and that of the 2nd cycle, since that the irr-CEs after the 1st cycle are quite similar to each other, indicating an additional irreversible reaction in the 1st cycle, we attributed it to the 1st cycle extra SEI formation due to the initial reductive electrolyte decomposition on the Cu surface when the cell is first discharged, namely 1st extra SEI in the protocol.

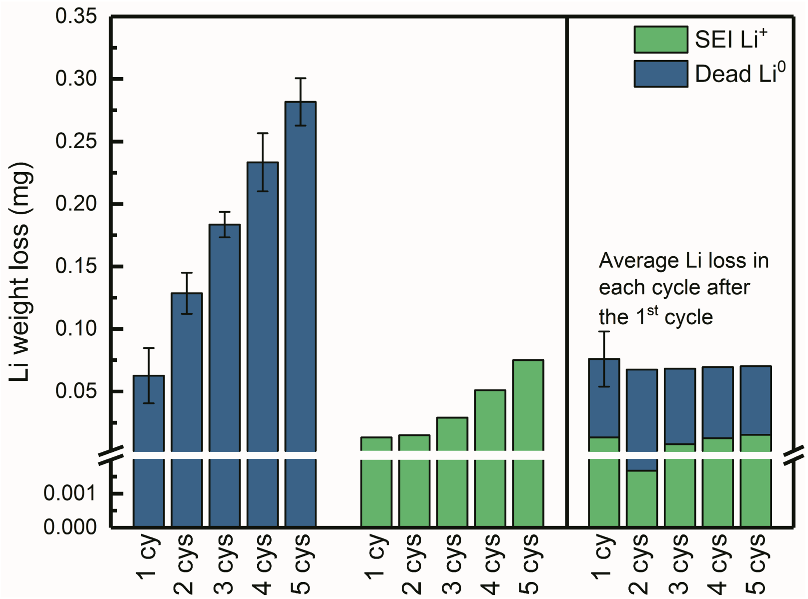

Supplementary Fig. 2. TGC measurements quantifying the amount of dead Li^0 and SEI Li^+ associated with the total capacity loss of Li//Cu cells after one, two, three, four, and five cycles (the 1st, 2nd, 3rd, 4th, 5th).

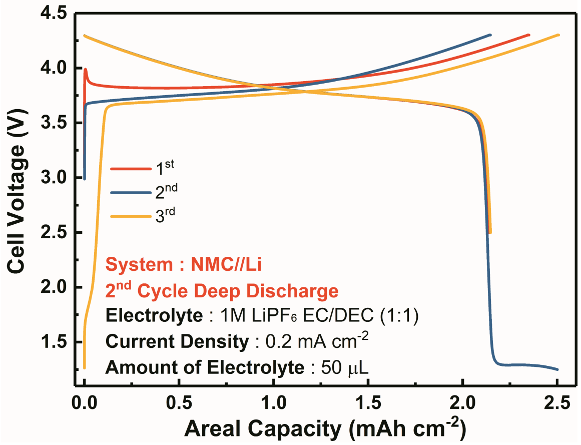

Supplementary Fig. 3. Charge and discharge curves of NMC//Li cell with deep discharge in the 2nd cycle and normal discharge in the 1st and 3rd cycles, respectively.

Calculation of average coulombic efficiency of NMC//Li and NMC//Cu cells

For NMC//Li cells,

$$\frac{\mathrm{Capacity_{(n)}}}{\mathrm{Capacity_{(1)}}} = \mathrm{Average\ CE^{(n-1)}}, \text{ when n > 2 and A/C > 1.}$$

$$\ln\left(\frac{\mathrm{Capacity_{(n)}}}{\mathrm{Capacity_{(1)}}}\right) = (\mathrm{n}-1)\ln(\mathrm{Average\ CE}) \text{............Equation (1)}$$

Therefore, the slope of the $\ln\left(\frac{\mathrm{Capacity_{(n)}}}{\mathrm{Capacity_{(1)}}}\right)$ vs. $(\mathrm{n}-1)$ is $\ln(Average\ CE)$.

As for NMC//Cu cells, we calculated the average CE after the transition state, namely in A/C ratio < 1 region.

$$\frac{Capacity_{(n)}}{Capacity_{(t)}} = Average\ CE_{transition}^{(n-t)} \text{, when n > t and A/C < 1.}$$

$$\ln\left(\frac{\mathrm{Capacity_{(n)}}}{\mathrm{Capacity_{(t)}}}\right) = (\mathrm{n}-\mathrm{t})\ln(\mathrm{Average\ CE_{transition}}) \text{............Equation (2)}$$

Therefore, the slope of the $\ln\left(\frac{\mathrm{Capacity_{(n)}}}{\mathrm{Capacity_{(t)}}}\right)$ vs. $(\mathrm{n}-\mathrm{t})$ is $\ln(\mathrm{Average\ CE_{transition}})$.

Procedures to obtain irr-CEs in different cell configurations

In this section, the detailed step-by-step flowchart of the proposed protocol is provided as shown in Supplementary Fig. 4. We first dissect the irr-CEs in Li//Cu cell by subtracting the 1st cycle irr-CE from the 2nd cycle irr-CE to quantify the contribution of 1st extra SEI formation. Secondly, the irr-CE remained and those of the following cycles can be attributed to dead Li and the subsequent SEI formation (dead Li + sub. SEI) due to the reductive electrolyte decomposition. On the other hand, the 1st cycle irr-CE of cathode//Li cell can be explained as the 1st irr-capacity of cathode together with the oxidative electrolyte decomposition (Ox. E.D.). As for the subsequent cycles, the origins of irr-CEs can be separated into two sources. When the reversible capacity remains the same and stable, the irr-CE of the cell can be attributed to the subsequent oxidative electrolyte decomposition (Sub. Ox. E.D.); however, when the reversible capacity starts to decline, then the irr-CE would become the sum of cathode degradation (cathode degrad.) and sub. Ox. E.D. due to the fact that capacity fading is directly related to the cathode degradation. To be more specific, the fraction of cathode degradation can be calculated from the slope of the fitted line of the normalized discharged capacity retention based on equation (1); thus, the fraction of sub. Ox. E.D. within the capacity fading region could be quantified as the difference between the total irr-CE and that of cathode degradation.

After identifying and quantifying the irr-CEs in Li//Cu and cathode//Li cells, the obtained irr-CEs can be further transferred into the NMC//Cu cell (AFLMB) at its different A/C ratio states. Thus, identifying the A/C ratio of the cathode//Cu cell is crucial for the proposed protocol and could guide us through the steps in the flowchart to dissect the irr-CEs in cathode//Cu cell comprehensively (Supplementary Fig. 4). Hence, we propose to consider and quantify the loss of CE and capacity from a new aspect, i.e., the anode/cathode (A/C) ratio, in the AFLMBs.

When the A/C ratio is already less than one in the 1st cycle of cathode//Cu cell, the 1st cycle irr-CE is controlled by anode electrode and can be separated into three components, namely 1st extra SEI formation, dead Li + sub. SEI, and cross-talk effects. The fraction of 1st extra SEI and dead Li + sub. SEI can be transferred from the 1st cycle irr-CE in Li//Cu cell; thus, that of cross-talk effects can be calculated by subtracting the sum of the 1st cycle irr-CE in Li//Cu cell from the total 1st cycle irr-CE in cathode//Cu cell. As in the subsequent cycles, the irr-CE of NMC//Cu cell can be dissected into dead Li + sub. SEI (transferred from Li//Cu cell) and the cross-talk effects (calculated from the irr-CE difference between Li//Cu and NMC//Cu cell in the subsequent cycles).

Meanwhile, if the initial A/C ratio is larger than one, namely cathode is the limiting electrode, then the 1st cycle irr-CE is dominated by cathode and equals to the fraction of the 1st irr-capacity of cathode. In the subsequent cycles, the irr-CE

can be separated into cathode degradation and sub. Ox. E.D. when A/C ratio remains larger than one, namely before the transition state. It should be noted that the fraction of cathode degradation in cathode//Cu cell could also be calculated from equation (1) like in cathode//Li cell; however, the value may not necessarily equal to that in cathode//Li cell considering the cathode degradation mechanism may be different among two cell configurations. However, as the active Li suffers from the continuous consumption by dead Li and subsequent SEI formation, the A/C ratio would eventually become less than one. Therefore, the anode electrode is then the limiting electrode and dominates the irr-CE of cathode//Cu cell, which is comparable to the aforementioned A/C < 1 case that the irr-CE can be separated into cross-talk effects and dead Li + sub. SEI.

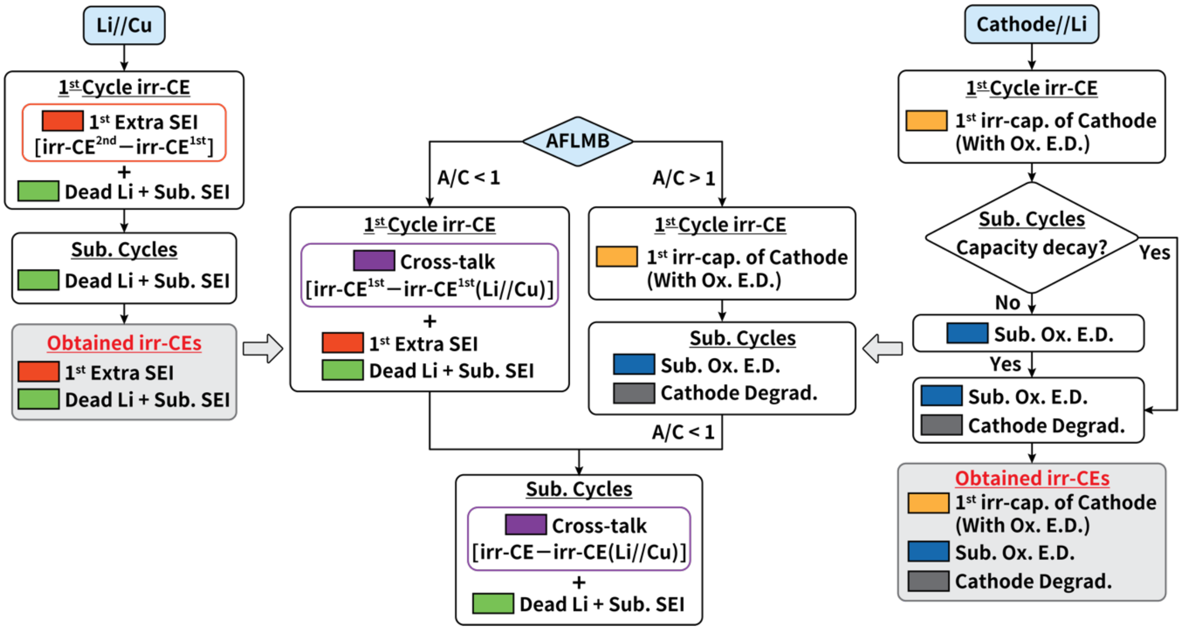

Supplementary Fig. 4. A step-by-step flowchart of the proposed protocol for identifying the origin of each irr-CE in different cell configurations.

Experimental validation for the proposed protocol.

In order to further validate the reliability of the results obtained from the proposed protocol and confirm if dead Li is the dominant source of irr-CE of AFLMB, the amount of dead Li at different cycles of AFLMBs and Li//Cu cells at their Li-stripped state is measured using the TGC methods reported by Meng et al.[1] and compared with the values dissected from the protocol as shown in Supplementary Fig. 5. It is observed that in Li//Cu cell, the deduction of the 1st extra SEI formation from the 1st cycle irr-CE is close to the dead Li obtained by titration gas chromatography (TGC) method, which confirms the reliability of our proposed protocol for quantifying the origins of irr-CE in Li//Cu cell. However, it should be noted that since the SEI fracture and formation would continue to occur in the subsequent cycles due to the unstable property of the as-formed SEI and dendrite formation[2]. Thus, it is also observed that the fraction of sub. SEI formation increased in the subsequent cycles.

Meanwhile, it should be noted that for the validation of NMC//Cu cells, Li^0 is used to represent the origin of the obtained value from the TGC method instead of dead Li since the presence of excess active Li after the 1st cycle of NMC//Cu cell left on Cu which is not truly “dead”. From the results shown in Supplementary Fig. 5, there is also a discrepancy between the total irr-CE value and the sum of 1st extra SEI formation and Li^0 amount from the TGC method. This difference could originate from sub. SEI formation and the cross-talk effects in the 1st cycle of the NMC//Cu cell. As in the 2nd and 10th cycles, the increased discrepancy between the total irr-CE value and that of measured by the

TGC method can be again explained by the subsequent SEI formation and cross-talk effects. To conclude, the results from the validation are in consistent with those obtained from the integrated protocol, namely the major source of irr-CE in AFLMB should be attributed to the formation of dead Li due to poor reversibility of Li plating and stripping. Lastly, for further evaluation of the protocol under different circumstances, two example studies are conducted by changing the current density and electrolyte formulation in the following sections, respectively.

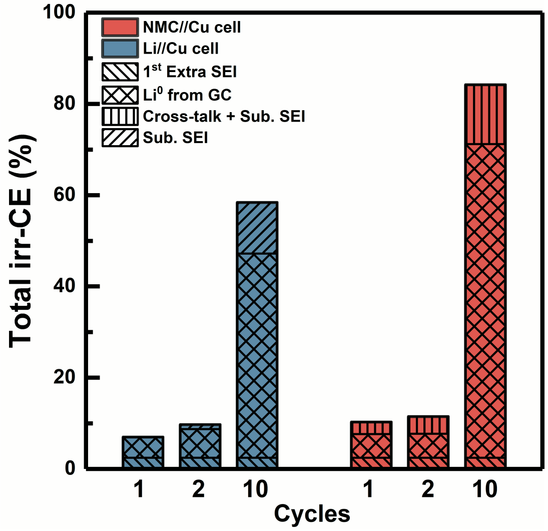

Supplementary Fig. 5 Inactive Li validation of Li//Cu and NMC//Cu cells for the irr-CE obtained from the proposed protocol using the titration gas chromatography method.

Preparation of different amount of Lithium Metal

The A/C = 50 and A/C = 16.5 Li were calculated directly from a 300 μm and 100 μm Li foil (16 mm in diameter), respectively. The A/C= 10 and A/C = 2 pre-plated Li was prepared by applying 0.1 mA cm^{-2} on a copper foil (19mm in diameter) to plate Li metal from a Li foil with the diameter of 16mm for 135 and 27 hours, respectively.

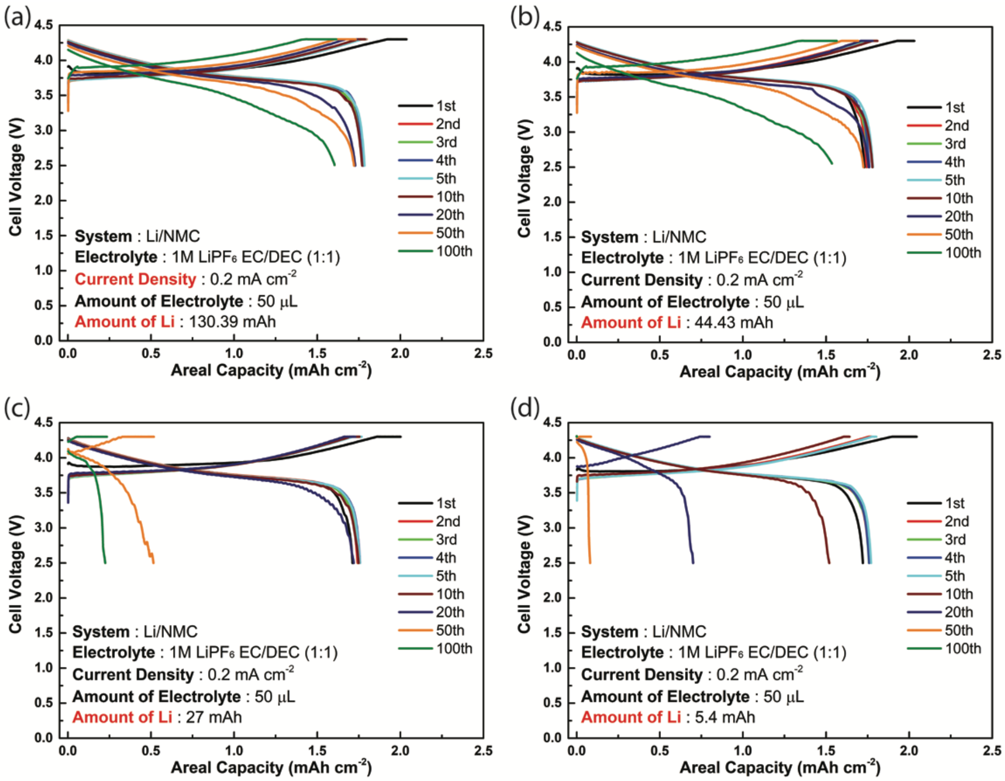

Supplementary Fig. 6. Charge/Discharge profiles of NMC//Li cells with (a) A/C = 50, (b) A/C = 16.5, (c) A/C = 10, and (d) A/C = 2.

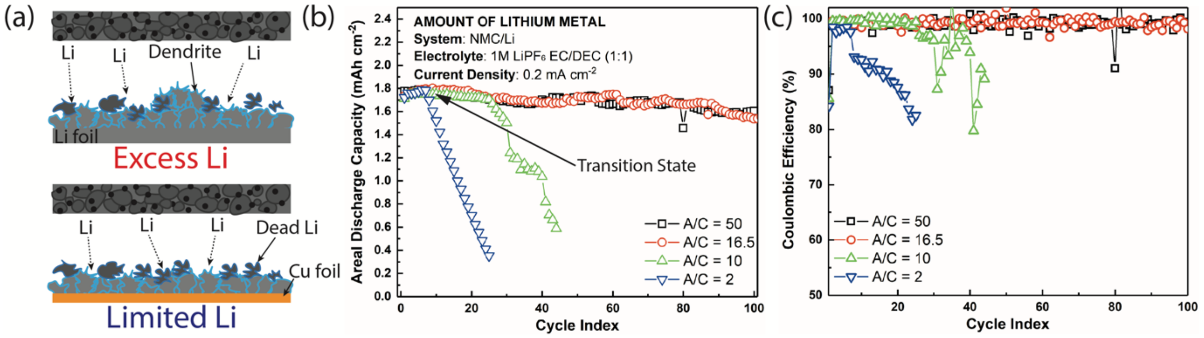

Supplementary Fig. 7. Scheme, cycle life, and coulombic efficiency comparison of cells using different amounts of Li at the negative electrode.

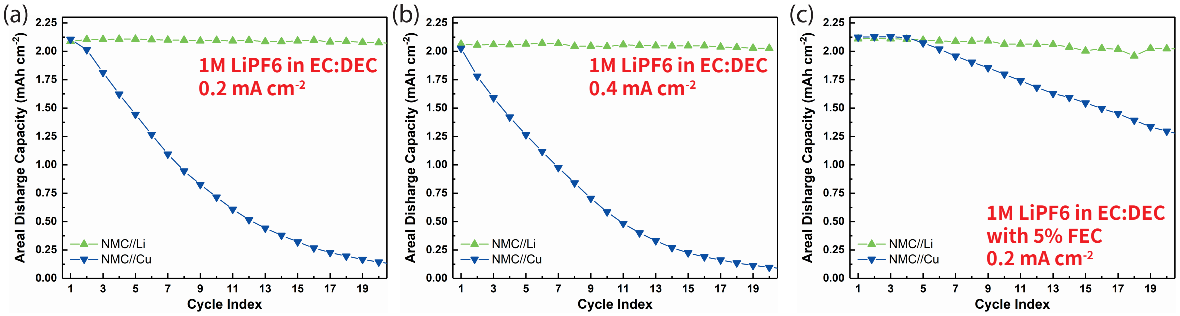

Supplementary Fig. 8. Areal capacity retention under different parameters. (a) 0.2 mA cm^{-2} of current density, (b) 0.4 mA cm^{-2} of current density, and (c) 0.2 mA cm^{-2} of current density with 5% FEC added in commercial electrolyte, 1M $LiPF_6$ in EC:DEC.

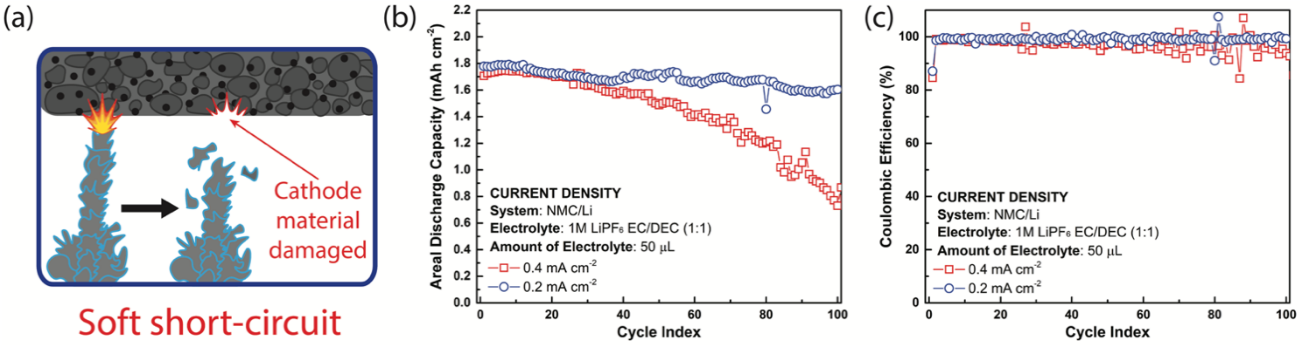

Supplementary Fig. 9. (a) Scheme of soft short-circuit, (b) areal discharge capacity, and (c) coulombic efficiency of NMC/Li cells under different current density.

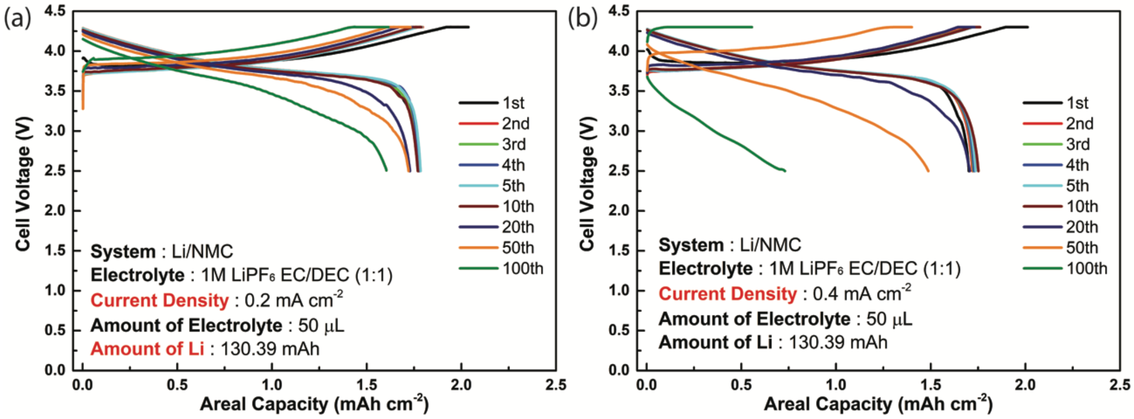

Supplementary Fig. 10. Charge/Discharge curves of NMC/Li cells under (a) 0.2 mA cm^{-2} and (b) 0.4 mA cm^{-2} of current density.

Comparison of Initial Overpotential and Polarization at 50% SOC

Supplementary Fig. 11 shows the corresponding initial overpotential, the polarization of Li//Cu, NMC//Li, and NMC//Cu cells under different parameters.

For a low current density of 0.2 mA cm^{-2}, Li//Cu cell shows larger initial overpotential and polarization at 50% SOC than Li//Li (Fig. S6a). This can be explained by the lower lithiophilicity of Cu, which leads to a larger Li nucleation barrier on Cu. It is also noticed that during the stripping process of Li//Cu cell, different from Li//Li cell, the second

plateau relating to the Li^+ diffusion from the bulk Li beneath the thick SEI formed due to the low reduction potential of metallic Li after cell assembly. This can be attributed to the substitution of Cu for Li, in which bulk Li does not exist thus the thick SEI is absent. In addition, it is found that the initial nucleation overpotential of Li//Li cell is strongly influenced by the length of resting time before cycling as shown in Fig. S1, the longer the cell rests, the higher the initial nucleation overpotential. As a result, when applying the proposed protocol, the resting time of each cell should also be considered and controlled.

Lastly, when using 5% of FEC as an additive in the commercial electrolyte, the initial overpotential and polarization at 50% SOC are both smaller than that in the electrolyte without 5% FEC except Li//Li cell, suggesting a more stable yet Li^+ conductive SEI formed after adding FEC into the electrolyte, which reduces the charge transfer resistance between the electrode/electrolyte interface.

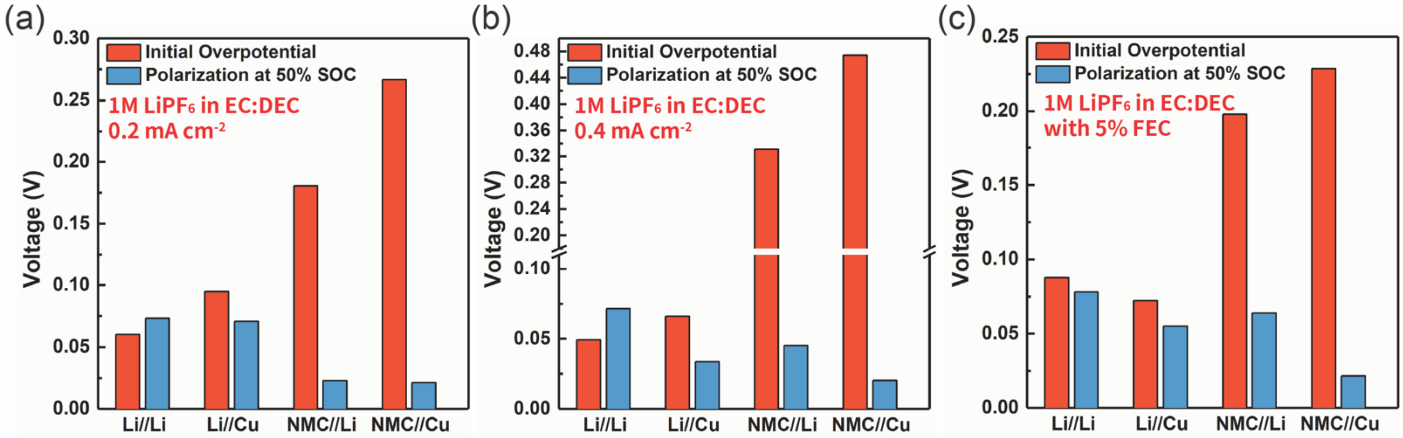

Supplementary Fig. 11. Initial overpotential and polarization at 50% state of charge (SOC) comparison. (a) 0.2 mA cm^{-2} of current density, (b) 0.4 mA cm^{-2} of current density, and (c) 0.2 mA cm^{-2} of current density with 5% FEC added in commercial electrolyte, 1M $LiPF_6$ in EC:DEC. The initial overpotential of NMC//Li and NMC//Cu cells are calculated by the initial cell voltage difference of the 1st and the 2nd cycle.

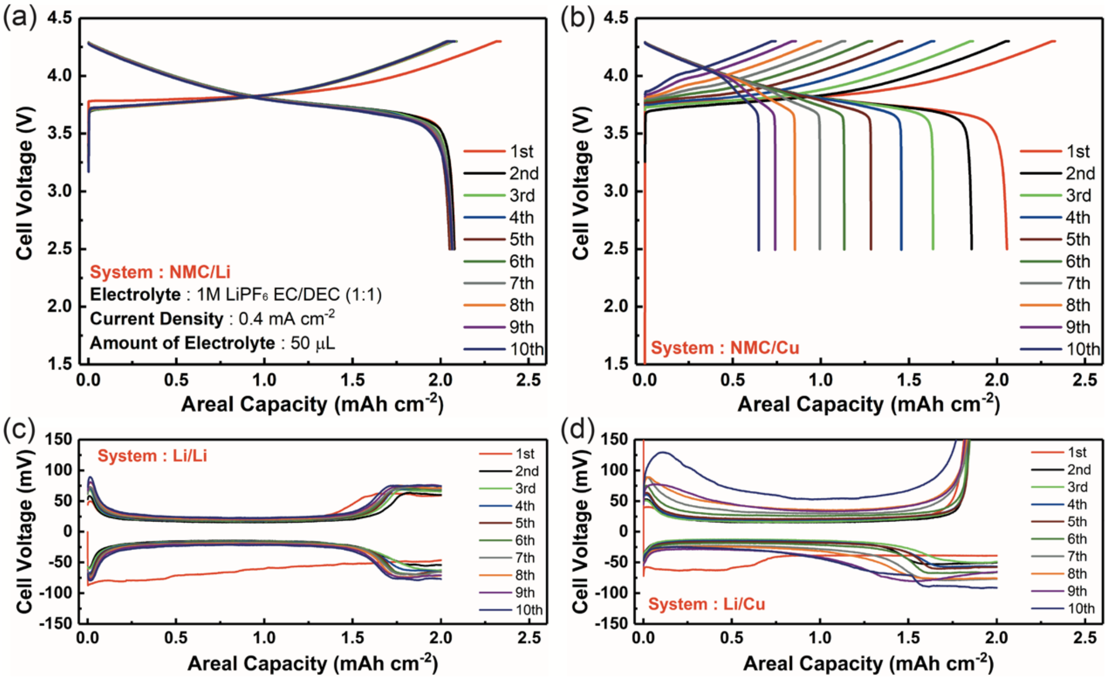

Supplementary Fig. 12. performance of integrated protocol, (a) NMC/Li, (b) NMC/Cu, (c) Li/Li, and (d) Li/Cu, using 1M $LiPF_6$ in EC:DEC as electrolyte with the current density of 0.4 mA cm^{-2}. We selected NMC532 as the cathode material used in this electrolyte, one can substitute desired cathode material for cathode study in half-cell and AFLMB.

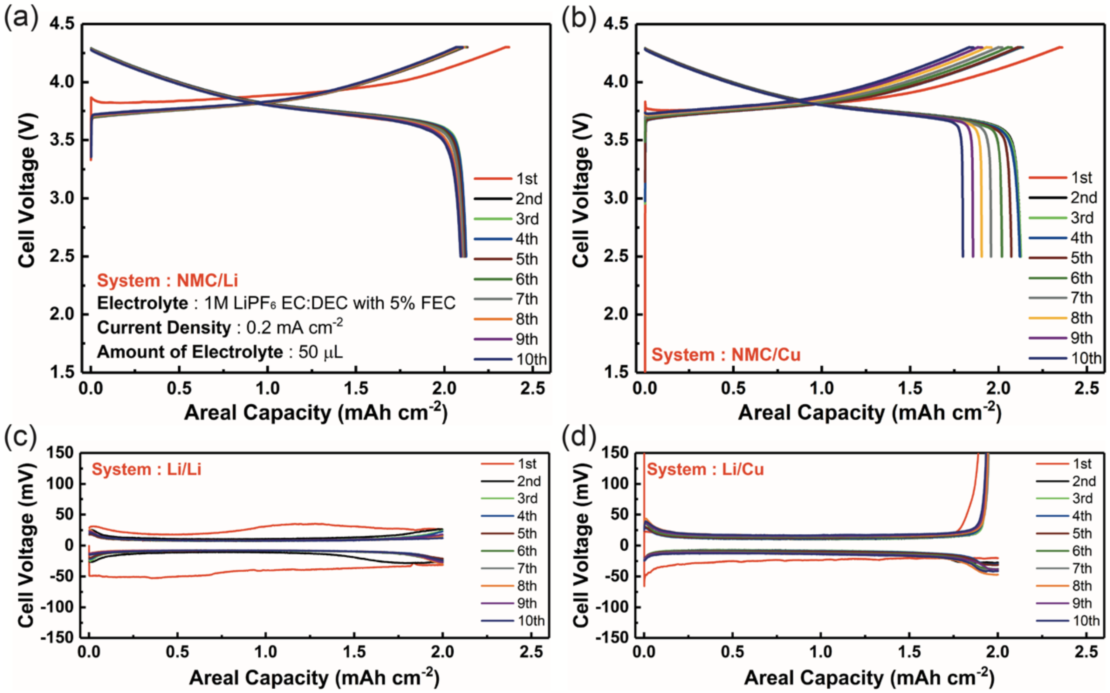

Supplementary Fig. 13. Electrochemical performance of integrated protocol, (a) NMC/Li, (b) NMC/Cu, (c) Li/Li, and (d) Li/Cu, using 1M $LiPF_6$ in EC:DEC with 5% of FEC as electrolyte with the current density of 0.2 mA cm^{-2}. We selected NMC532 as the cathode material used in this electrolyte, one can substitute desired cathode material for cathode study in half-cell and AFLMB.

Extending the proposed protocol to ether system

We further extended our proposed protocol for evaluating the performance of AFLMB within the ether systems. 1M LiTFSI in DME:DOL (1:1) with 2 wt% of $LiNO_3$ added and lithium iron phosphate ($LiFePO_4$, LFP) are selected as the electrolyte and cathode material for demonstration, respectively. For the 1st cycle irr-CE of Li//Cu cell (Supplementary Fig. 14b), the total irr-CE of 1.91% can be separated into 0.31% of 1st extra SEI formation and 1.60% of dead Li + sub. SEI. Subsequently, the irr-CE at the 2nd cycle is 1.60% and keeps around ~1% in the subsequent cycles, which can be attributed to dead Li + sub. SEI. Meanwhile, LFP//Li cell shows the 1st cycle irr-CE of 2.93% for 1st cathode irreversible capacity. It is noted that the irr-CEs after the 1st cycles are mostly greater than 1%, which is higher than those in carbonate systems and can be explained as greater amount of oxidative electrolyte decomposition in ether-based system than that in carbonate-based electrolyte due to the lower upper limit of potential window for ethers. Moreover, the fraction of cathode degradation in the irr-CE in the subsequent cycles can be calculated from the normalized capacity retention, which is ~0.17% in each cycle (see the gray bar in Supplementary Fig. 14b). Last but not the least, the 1st cycle irr-CE in LFP//Cu cell is 11.30%, which is much higher than that of 1.91% in Li//Cu cell and 2.93% in LFP//Li cell. However, it is confirmed that the capacity loss in the 1st cycle mainly originated from the loss of active Li inventory, since the 1st cycle lost capacity of LFP could be restored after re-assembling the cycled LFP electrode with metallic Li anode (see Supplementary Fig. 15a). Based on the result, we suggest that the initial A/C ratio of LFP//Cu cell is less than one due to higher capacity loss at the anode and the irr-CE should be dominated by the anode. Thus, apart from the 0.31% of 1st extra SEI formation and 1.60% of dead Li + sub. SEI, the remained 9.39% of irr-CE is suggested to be cross-talk effects. In the subsequent cycles, the irr-CEs in LFP//Cu cell are still larger than those in Li//Cu cells, suggesting cross-talk effects hold a remarkable proportion of irr-CE in LFP//Cu cell when using 1M LiTFSI in DME:DOL (1:1) with 2 wt% $LiNO_3$ added as the electrolyte. Thus, the irr-CE in the 2nd cycle can be attributed to 1.6% dead Li + sub. SEI and 2.56% cross-talk effects, and that in the 20th cycle to 1.06% dead Li + sub. SEI and 1.01% cross-talk effects. In addition, we reassembled the cycled LFP electrode (from the dead LFP//Cu cell) with metallic Li anode to confirm the origin of capacity loss in LFP//Cu cell (Supplementary Fig. 15b). The results show that the reversible capacity of LFP//Li cell is regained back to the level of the 1st charge capacity, thus, suggesting the capacity loss in LFP//Cu cell is mainly due to the irreversible reactions from anode, namely dead Li and SEI formation and the cross-talk effects. From the information obtained from the integrated protocol, it can be concluded that dead Li formation is significantly suppressed in ether-based electrolyte with the addition of $LiNO_3$, which is similar to the previous works reporting the stabilizing effect of $LiNO_3$ to metallic Li anode[3]. Thus, the capacity decay rate of the anode-free LFP//Cu cell is remarkably retarded compared to that of NMC//Cu cells in carbonate systems.

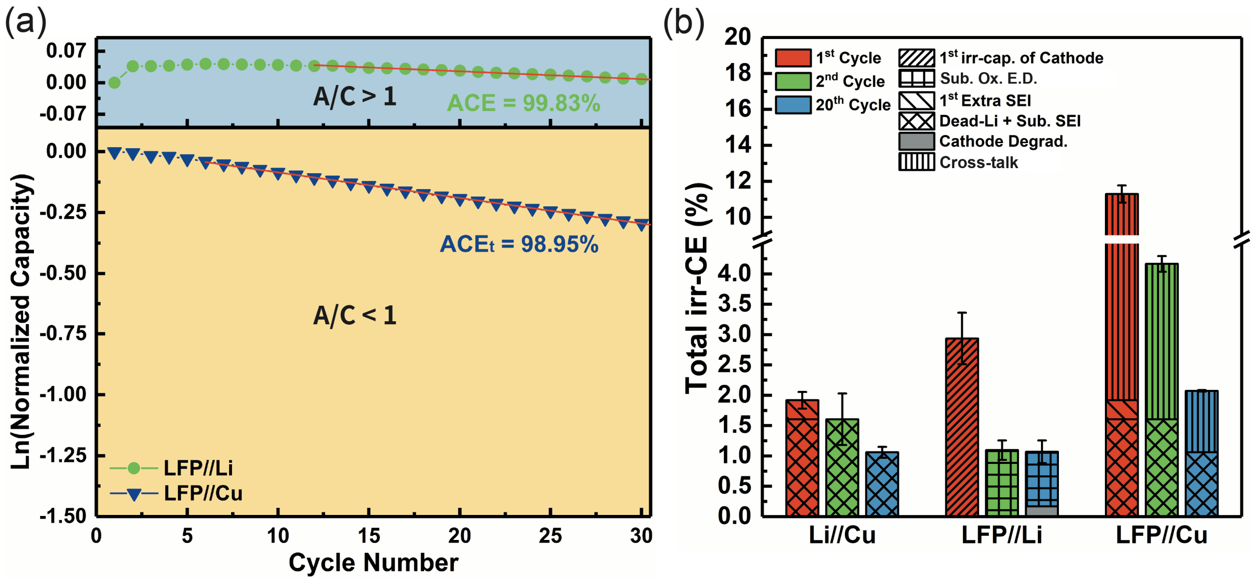

Supplementary Fig. 14. Results obtained from the integrated protocol using 1M LiTFSI in DME:DOL (1:1) with 2 wt% of $LiNO_3$ added as electrolyte under the current density of 0.17 mA cm^{-2} (0.1 c-Rate to the LFP electrode). **a**, Normalized discharge capacity versus cycle number of LFP//Li and LFP//Cu cells. **b**, irreversible CE comparison of Li//Cu, LFP//Li, and LFP//Cu cells at different cycles. Error bars represent standard deviation, n = 3 independent replicates.

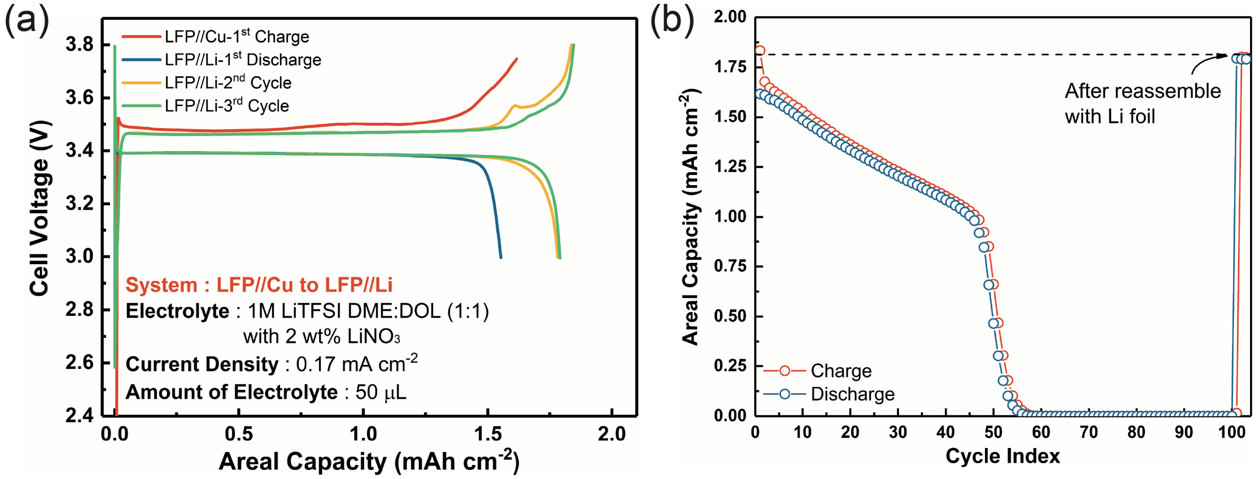

Supplementary Fig. 15. a, Charge/discharge curves of LFP//Cu in the 1st charge process and reassemble the LFP electrode with metallic Li anode for discharge process and the subsequent cycles. **b**, Areal capacity retention of LFP//Cu cell and the reassembled LFP//Li cell using the same LFP electrode in LFP//Cu cell.

Reference:

1. Fang C, *et al.* Quantifying inactive lithium in lithium metal batteries. *Nature* **572**, 511-515 (2019).

2. Thirumalraj B, *et al.* Nucleation and Growth Mechanism of Lithium Metal Electroplating. *J Am Chem Soc* **141**, 18612-18623 (2019).

3. Li W*, et al.* The synergetic effect of lithium polysulfide and lithium nitrate to prevent lithium dendrite growth. *Nat Commun* **6**, 7436 (2015).
