# Supplementary material for: Decoupling the origins of irreversible coulombic efficiency in anode-free lithium metal batteries
Source: Nat Commun. 2021 Mar 4;12:1452. doi: 10.1038/s41467-021-21683-6 (PMC7933276; doi:10.1038/s41467-021-21683-6)
Supplement: Supplementary file 3 — Description of Additional Supplementary files [file 41467_2021_21683_MOESM3_ESM.docx]

**Description of Additional Supplementary Files**

File name: Supplementary Movie 1

Description: In-situ OM observation of lithium plating process.

File name: Supplementary Movie 2

Description: In-situ OM observation of cell shorting.

File name: Supplementary Movie 3

Description: In-situ OM observation of lithium stripping process.

File name: Supplementary Movie 4

Description: In-situ TXM observation of lithium plating process.

File name: Supplementary Movie 5

Description: In-situ TXM observation of lithium stripping process.
